# Impact of Warning Pop-Up Messages on the Gambling Behavior, Craving, and Cognitions of Online Gamblers: A Randomized Controlled Trial

**DOI:** 10.3389/fpsyt.2021.711431

**Published:** 2021-07-23

**Authors:** Julie Caillon, Marie Grall-Bronnec, Anaïs Saillard, Juliette Leboucher, Morgane Péré, Gaelle Challet-Bouju

**Affiliations:** ^1^Addictology and Psychiatry Department, CHU Nantes, Nantes, France; ^2^INSERM, Methods in Patient-Centered Outcomes & Health Research U1246 ≪Biostatistics, Pharmacoepidemiology and Human Science Research≫, Nantes University, Tours University, Nantes, France; ^3^Biostatistics and Methodology Unit, Department of Clinical Research and Innovation, CHU Nantes, Nantes, France

**Keywords:** internet gambling, problem gambling, responsible gambling, pop-up message, prevention, addiction

## Abstract

**Background:** Many features of Internet gambling may impact problem severity, particularly for vulnerable populations (availability, anonymity, a convenience and ease of play, digital forms of payment, and a higher level of immersion). To prevent the risks associated with excessive gambling and to inform gamblers, we need responsible gambling strategies. Gambling-related warning messages are one possible strategy that can help minimizing gambling-related harm.

**Methods:** Our experimental study aimed to evaluate the effectiveness of self-appraisal and informative pop-up messages compared to a control condition (blank pop-up messages), for both at-risk (ARG) and low risk/non-problem Internet gamblers (LR/NPG) according to their favorite type of game, in a semi naturalistic setting and with a 15-day follow-up. During the experimental session, participants were invited to gamble on their favorite website with their own money in the laboratory. Effectiveness was investigated through the impact of pop-ups on gambling behavior (money wagered and time spent), craving, cognitive distortions, and gambling experience, taking into account message recall. We analyzed data from 58 participants, playing preferentially either to skill and chance bank games (sports betting, horse race betting) and skill and chance social games (poker).

**Results:** We observed a significant decrease in the illusion of control for ARG in the informative pop-up condition at the 15-day follow-up. A significant effect of self-appraisal pop-ups compared to blank pop-up messages was also demonstrated only for sport and horse bettors, with a decrease on time spent gambling and an increase of gambling-related expectancies at the follow-up. Finally, we also observed that a majority of the participants were disturbed and irritated by pop-ups during their gambling session.

**Conclusions:** The results of our study demonstrated the limited impact of pop-up warning messages on gambling behavior and cognition in Internet gamblers according to the type of game and the status of gamblers. The limited impact of warning messages on gambling behavior and the inconvenience of the pop-ups for Internet gamblers lead us to only consider warning messages as one piece of a larger responsible gambling strategy.

**Trial Registration Number:** NCT01789580 on February 12, 2013.

## Background

Gambling is a popular activity that can lead to negative consequences such as spending more time or money on gambling than gamblers can afford, causing significant distress, and/or resulting in the loss of a significant relationship or job (1). A recent study examined the association between the part of income dedicated to gambling and 31 financial, social, and health outcomes for 6.5 million individuals over a period of 7 years (2). Gambling was associated with several harm, including higher financial distress, negative well-being, higher rates of future unemployment, and even increased mortality at the highest levels. Many features of Internet gambling may impact problem severity, particularly for vulnerable populations such as constant availability, anonymity, a convenience, and ease of play; digital forms of payment; and a higher levels of immersion (3–5). Epidemiological studies are unclear and the relationship between Internet gambling and addictive behavior has not been confirmed (4, 6, 7). However, studies observed that Internet gambling is more frequent among highly involved gamblers, and Internet significantly contributes to gambling problems (4, 8, 9).

To prevent the risks associated with gambling practice, strategies to promote responsible online gambling were developed. The aim of responsible gambling programs is to reduce the prevalence and incidence of gambling-related risks by helping individuals gamble appropriately within their means (10). Consequently, these strategies aim to impact vulnerable gamblers without disrupting all gamblers (11). These strategies especially include several types of harm-minimization tools, such as self-exclusion programs, self-limitations on money wagered and time spent, or warning messages, that aim to prevent or reduce gambling-related harm (12–14).

Initially created to inform the consumer about the risks associated with tobacco and alcohol consumption (15), warning messages to prevent gambling risks were developed internationally several decades ago (16). Several studies have evaluated the effectiveness of gambling-related warning messages (16, 17). The majority of them used electronic gambling machines (EGM) as the form of gambling studied, and only few of them evaluated the effectiveness of warning messages on Internet gambling (16, 18). Their results demonstrated that messages could inform gamblers and potentially produce changes in gambling behavior if applied appropriately. Dynamic, brief, and easy-to-read messages that appeared in the middle of the screen were the most effective display mode (16, 17, 19, 20). Pop-up messages that interrupted gambling were a good way to reduce dissociation (21) with a low negative effect on the gambling experience (18, 22). Furthermore, several studies demonstrated that the content of the warning messages is important for impacting gambling behavior (23). It seems that informative messages about the risks associated with gambling and gambling-related cognitive distortions (e.g., *Only spend what you can afford to lose*) increase gamblers' ability to stop gambling compared to blank messages (24). Other studies concluded that self-appraisal messages that were based on personalized feedback and that encouraged self-reflection about gambling behavior or limit-setting (e.g., *Are you gambling longer than planned?)* have a larger impact than informative pop-up messages (25, 26). Self-appraisal messages encourage gamblers to change their behavior by making reference to the individual's life, needs, and problems (18). As underlined by Monaghan and Blaszczynski, self-appraisal messages were recalled most often (27). Message recall is often necessary for the highest message impact in terms of persuasion resulting in desired behavioral changes (18). Despite the large body of evidence that supports the positive effect of pop-up messages on responsible gambling, a recent study concluded that many gamblers do not read the content of pop-up messages, thus questioning the possibility that informative or self-appraisal messages can be effective in reducing gambling behavior (28).

Given the discrepancies highlighted in previous research and because of potentially negative consequences associated with Internet gambling, new research is needed to further clarify the effectiveness of warning messages on this type of game. As underlined by Ginley et al. (16), many studies took place in a laboratory with a simulator, but the results could be different in real-world gambling environments (29). In the few studies conducted in real-world gambling environments (16), the sample size was generally large, and the methodology was robust. However, it could be technically difficult to test different modalities of a responsible gambling tool in the real world, and the possibilities for collecting data, particularly qualitative data, are generally more limited than in a laboratory. Moreover, the majority of the published studies included a large population of gamblers but did not differentiate between or compare non-problem gamblers and at-risk or problem gamblers. Finally, little is known about the effectiveness of pop-up warning messages in different types of games. In their study, Griffiths and Auer underlined the need to control this variable (25).

Thus, it seemed necessary to develop a controlled experimental study in a semi naturalistic setting to evaluate the effectiveness of Internet pop-up warning messages for different types of games and gambler statuses, and to collect both quantitative and qualitative data about gamblers' experience. During the experimental session, participants were invited to gamble in the laboratory on their favorite website with their own money. The objective of our experimental study was to evaluate the effectiveness of self-appraisal and informative pop-up messages compared to a control condition in both at-risk and non-problem Internet gamblers, taking into account the type of game, and assessing both immediate effect and medium-term effect. Effectiveness was investigated through the impact on gambling behavior, craving, cognitive distortions, and gambling experience, taking into account message recall. We hypothesized that self-appraisal pop-up messages that encourage participants to take a break and consider changing their gambling behavior might have a larger impact than informative pop-up messages and blank pop-up messages. We also hypothesized that the effectiveness of pop-up messages would vary according to the type of game and the status of gamblers.

## Methods

This study is part of an experimental randomized controlled trial targeted risk prevention conducted in both non-problem and at-risk gamblers (ARGs) (MOD&JEU study, ClinicalTrials.gov Identifier: NCT01789580). This trial aimed to determine the effectiveness of four types of gambling moderators: limiting bonuses, self-limitations, pop-up messages, and self-exclusion programs [for more detail, see Caillon et al. (30)].

### Participants

Participants were volunteers gambling regularly and currently on the Internet. We included ARGs [scoring 3–7 on the Problem Gambling Severity Index (PGSI) (31)] and low risk/non-problem gamblers (LR/NPG) (scoring 0–3 on the PGSI) of both sexes. Excessive gamblers (scoring 8 or higher on the PGSI) were not included because it would not have been ethical to expose them to the procedure of the study, which included a real gambling session. Other inclusion criteria were (i) being 18 or older (gambling is forbidden to minors in France), (ii) having gambled at least once in the past month in an authorized online gambling activity (in France, only lotteries, scratch card games, horse betting, sports betting, and poker are legalized forms of online gambling) on a website licensed by the ARJEL (an independent administrative governmental authority specifically dedicated to regulating online gambling), and (iii) agreeing to provide access to their gambling account to the research team to collect gambling history during the experiment. Non-inclusion criteria were (i) being treated for a gambling problem at the time of the experiment, (ii) being in debt, (iii) having used psychoactive substances on the day of the experiment, (iv) participating in another clinical study in the week preceding the experiment, being pregnant, (v) being under protection (guardianship or curatorship), or (vi) having a history of psychosis or cognitive impairment.

### Ethics

The participants were informed about the research and gave their written informed consent prior to their inclusion in the MOD&JEU study. This study was approved by the French Research Ethics Committee (CPP) on January 8, 2013. From an ethical point of view, given the gambling scenario, the protocol required reimbursement for the losses experienced by the participants during the gambling session, only if these losses exceeded the average amount of their usual losses. To avoid potential bias, participants were not informed of this procedure. Ultimately, no participants needed reimbursement for money wagered.

All participants received a 60€ gratification at the end of the post-test and 20€ after the follow-up for their participation in the study.

### Procedure

Participants were recruited through media announcements (newspapers, radio, and websites). In addition, we subcontracted recruitment to survey institutes to obtain lists of potential participants. Volunteers were asked to contact the research team by email to obtain details about the study and to arrange a telephone appointment to complete the pre-selection questionnaire that collected information about the severity of gambling problems (PGSI), gambling account information (money wagered, time spent) and verification of eligibility criteria. Recruitment for this study began in November 2015 and ended in March 2018.

Eligible participants completed a pre-test interview prior to the beginning of the experiment (T0) to collect the following information: sociodemographic data, gambling characteristics, severity of cognitive distortions [Gambling Related Cognitions Scale (GRCS) (32, 33)], craving [Gambling Craving Scale (GACS) (34, 35)], severity of gambling problems, use of online gambling protection measures and opinions on it, and gambling account information.

The participants were then randomly assigned to one of two experimental conditions [self-appraisal pop-up condition (*n* = 30), informative pop-up condition (*n* = 30), or control condition (*n* = 43)]. In order to optimize comparisons with the control group, the expected sample size of the control condition was 43, calculated as 30^*^√(*k*), where *k* was the number of experimental conditions. In each experimental condition, the number was set at 30 individuals, in order to obtain “large” groups in the statistical sense of the term, making it possible to obtain estimators of the normally distributed means (central limit theorem). In order to optimize all the comparisons, the groups were composed in orthogonal blocks taking into account the two factors of the preferred type of game (three levels) and the gambling status (two levels). The randomization was stratified using an algorithm designed by a biostatistician according to their favorite type of game [pure chance games (*n* = 35), skill and chance bank games (*n* = 34), or skill and chance social games (*n* = 34)] and to their gambling status [LR/NPG (*n* = 52) and ARGs (*n* = 51)].

In the three conditions, the participants were invited to come to our laboratory at the Nantes University Hospital to participate in an online gambling session. This gambling session was performed on the participant's favorite gambling website, with her/his own gambling account and with her/his own money, to reproduce a setting that was as naturalistic as possible. Participants were encouraged to gamble as usual, with instructions that they could stop at any time. The gambling session could last up to 3 h, and there was no minimum duration defined *a priori*. During the experiment, four pop-up messages appeared in the middle of the screen, causing a break in gambling until the gambler closed the window. The display frequency of the messages varied according to the usual gambling session duration communicated by the participant during the pre-test interview. For example, for sessions longer than 15 min, the session was divided into five periods, and a pop-up message was presented at the end of each period, except after the last one (never during a bet, imperatively between two bets). For sessions scheduled under 15 min, pop-ups were presented every 3 min, regardless of the total duration. After the final pop-up, participants could continue to play for as long as they wished. We were precisely able to control the pop-up presentation between bets because we could view the participant's screen and control it remotely on another screen. The pop-up message content varied according to the condition and was randomly allocated to the participants. Messages were brief and easy to read. The four informative messages were designed to inform participants of the nature and the risks of gambling, including potential negative consequences associated with gambling and offered content to correct cognitive distortions: “*Gambling involves risks: debt, loneliness, and addiction,” “When gambling, sometimes we lose not only money but also time,” “Play only with the money you can lose,”* and “*All gambling games are part chance.”* The four self-appraisal pop-ups were designed to encourage participants to take a step back and examine their own current gambling behavior: “*Do you know how long you have been playing?,” “Have you spent more money than you intended?,” “Do you need to think about taking a break?,”* and “*Are you trying to recover the money you lost previously while playing?.”* In the control group, pop-ups were displayed but did not contain a message.

Then, participants completed a post-test interview immediately at the end of the experiment (T1) and again 15 days after experiment completion (T2 by phone). The same information as in the pre-test was collected in the post-test interviews. Moreover, the impact of pop-up messages on gambling behavior was also questioned.

### Measures

#### Socio-Demographic Data

Age, sex, marital status, education level, and employment status.

#### Gambling Characteristics

Age of first gambling experience, gambling habits (type of game, frequency, and money wagered), and gambling motives.

#### Gambling Problems

Age at onset of gambling problems, and damages caused by gambling.

#### Gambling Severity

Problem Gambling Severity Index was used to evaluate the severity of gambling problems. This scale is a 9-item self-report questionnaire that indicates the status of the gambler: non-problem gambler (score 0), low-risk gambler (score 1–2), moderate-risk gambler (score 3–7), and problem gambler (score 8+). In the present study, the result of the PGSI was used to define two categories: ARGs (score 3–7), and LR/NPG (score of 0–2). We decided to regroup low risk gamblers with non-problem gamblers because it's possible for a regular and current gambler on the Internet to have a score of 1 or 2 without experiencing gambling problems.

#### Enjoyment of Gambling

At the end of the gambling session (T1 only), enjoyment of gambling was explored using a Likert-type scale from 0 (not at all) to 10 (extremely).

#### Impact of Pop-Up Messages on Gambling Behavior

Subjective effectiveness of warning messages on gambling behavior (experimental conditions only), display time of the pop-up message on the screen before being closed by the participant, and recall (free and cued recall) of the message content (experimental conditions only).

#### Gambling Account Information

We used participants' gambling history to gather objective information about money wagered and time spent gambling. The reference period considered concerned the last 7 active days (an active day was a day in which the participant gambled at least once) before the experimental session and before the follow-up. Gambling account information during the gambling session was also collected.

#### Severity of Cognitive Distortions

The 23-item GRCS was used to explore five dimensions of gambling-related cognitive distortions: interpretative bias (GRCS-IB), illusion of control (GRCS-IC), predictive control (GRCS-PC), gambling-related expectancies (GRCS-GE), and perceived inability to stop gambling (GRCS-IS).

#### Craving

The GACS was used to assess cravings for gambling (T2 only). This scale is a 9-item questionnaire in which participants were asked to indicate whether they agreed with each proposition on a 7-point scale (1: strongly disagree, 7: strongly agree). The structure of this questionnaire includes three dimensions, each represented by three items: “anticipation” (anticipating that gambling will be fun and enjoyable), “desire” (a strong and urgent desire to gamble), and “relief” (an expectation that gambling will provide relief from negative affect).

### Data Reduction

The main outcomes used to objectively estimate the impact of pop-up messages on gambling behavior were the raw value of money wagered and time spent gambling. We determined these variables by comparing the objective money wagered and time spent gambling during the experimental session (gathered from the gambling account information in the post-test) with the data of money wagered and time spent gambling collected in the pre-test. To evaluate the subjective effects of pop-up messages on gambling-related cognitions and behaviors, we computed change scores to express variations between the pre-test and post-test on GRCS and GACS scores. The enjoyment of gambling rating was used as is.

### Data Analysis

Statistical analyses were performed using SAS R software version 9.4 (SAS Institute Inc., Cary, NC, USA). Continuous variables were expressed as the mean ± standard deviation or as the median (25th−75th percentiles) when describing non-normal data. Categorical variables were expressed as a number (percentage). The normality of continuous variables was tested using the Shapiro-Wilk test, and transformations were applied whenever needed. Then, independent three-way ANCOVAs were performed to compare the money wagered and time spent gambling during the gambling experimental session between the two experimental conditions and the control condition, taking into account the gamblers' favorite type of game and status of gambler. The analyses included both the effect of the condition, the effects of the stratification variables (type of game and status of gambler), and the interaction between them. When the interactions were not significant, they were removed from the final models. When an effect was significant, pairwise comparisons were performed with Dunnett's tests only for the significant effects, by comparing each experimental condition to the control condition and controlling for the Type 1 experiment wise error. Supplementary independent three-way ANCOVAs were performed according to the same design to compare the enjoyment of gambling, and GRCS and GACS change scores.

## Results

**Figure 1:**
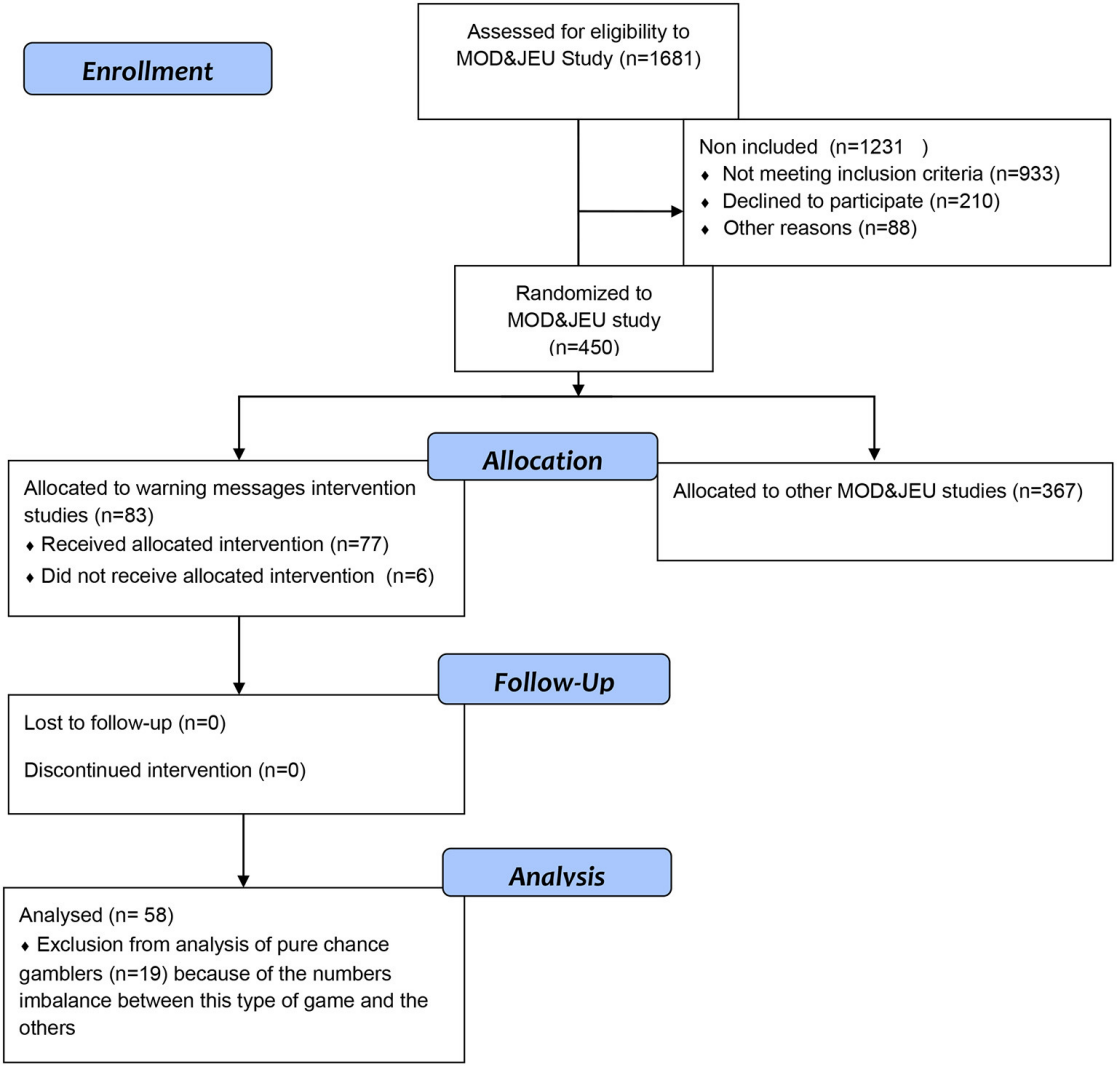
Flowchart of study.

Due to recruitment difficulties and given the numbers imbalance between this type of game and the others, pure chance gamblers (*n* = 19, including only one ARG) were excluded from the analyses. As a result, we analyzed data from 58 participants (Figure 1), including 26 gamblers of skill and chance bank games and 32 gamblers of skill and chance social games.

### Description of the Sample

#### Socio-Demographic Data

The majority of participants were men (78%) who were 37 years old on average. Two-thirds (64%) of the participants lived with a partner. Almost three quarters of participants were involved in a professional activity (73.4%), 21.8% did not work, and 4.7% were students.

#### Gambling Habits

The majority of participants (46.9%) gambled at least once per week, and 32.8% gambled every day or almost every day. On average, participants gambled 24.72€ per gambling session, and each session lasted approximately 63 min. According to their disposable income and leisure time availability, time spent and money wagered were considered appropriate for 90% of the participants.

One-third of participants (32.8%) gambled only online. All participants had previously gambled offline with money; the average age of the first gambling experience was 15 years old for offline gambling and 30 years old for online gambling.

The main online gambling motives were the fun (61%), the hope of making money (56.2%), the use of strategy (17.2%), and the ease of Internet gambling (accessibility, convenience) (15.6%).

While 62% (70.6% of LR/NPG vs. 53.3% of ARG) of the participants never felt that they had a problem with gambling, 22.2% experienced some kind of problem in the past, and 16% were experiencing problems at the time of the experiment. The majority of participants did not report any harm related to gambling on their life (76.5% of LR/NPG vs. 65.6% of ARG). The most frequently reported damages were time spent (50%), impact on mood like stress and anxiety (41%), and money spent (27%). The average age participants recognized they had a gambling problem was at the age of 32.2 years after online gambling initiation on average.

#### Opinion of the Pop-Up Harm-Minimization Tool Before the Experiment

The general opinion of the pop-up moderator was favorable for the majority of the participants (60% for informative pop-ups vs. 78% for self-appraisal pop-ups). Participants reported “*it's responsible to warn gamblers*,” “*it helps to think about one's own practice*,” and these messages had an “*educational purpose*.” Nevertheless, a significant portion of the participants had an unfavorable opinion of these types of message (40% for informative pop-up vs. 22% for self-appraisal pop-up). The most common explanation was that “*pop-ups could interfere with their gambling practice*.” Participants also thought these messages were “*useless because players would not read them.”*

### Comparison of the Experimental Groups and the Control Group

Table 1 describes the variables of interest according to conditions (control or experimental conditions), for the first (immediate) and second (15 days) post-tests.

**Table 1:** Description of the variables of interest according to the conditions (*n* = 58), for the 1st post-test (T1) and the 2nd post-test (T2).

	***Mean (SD)***		
	**Control condition** **(*****n*** **=** **23)**	**Informative pop-up** **(*****n*** **=** **16)**	**Self-appraisal pop-up** **(*****n*** **=** **19)**
**FIRST POST-TEST (T1, IMMEDIATE)**			
Money wagered (€)	13.15 (16.14)	5.19 (5.49)	21.45 (28.21)
Time spent (min)	58.57 (44.33)	39.31 (38.38)	47.00 (39.87)
Enjoyment of gambling (/10)	6.43 (2.06)	6.06 (2.62)	5.68 (2.08)
GRCS_GE change score (T1–T0)	−1.52 (2.54)	−1.13 (1.96)	−0.79 (1.55)
GRCS_IS change score (T1–T0)	−0.48 (3.17)	−0.50 (2.56)	0.00 (3.14)
GRCS_IC change score (T1–T0)	0.39 (2.17)	−0.75 (1.29)	−0.16 (1.74)
GRCS_PC change score (T1–T0)	−2.35 (3.47)	−1.44 (5.45)	−1.05 (3.46)
GRCS_IB change score (T1–T0)	−1.96 (3.30)	−2.13 (3.95)	−1.21 (3.68)
**SECOND POST-TEST (T2, 15 DAYS)**			
Money wagered (€)	176.73 (309.62)	154.19 (379.92)	416.20 (657.89)
Time spent (min)	686.25 (907.18)	383.00 (745.35)	482.11 (526.09)
GACS-Anticipation change score (T2–T0)	−0.81 (2.75)	−0.38 (2.19)	0.11 (2.31)
GACS-Desire change score (T2–T0)	−0.52 (1.73)	−0.31 (3.00)	0.32 (1.70)
GACS-Relief change score (T2–T0)	0.13 (1.63)	−0.94 (3.09)	−0.21 (2.76)
GRCS_GE change score (T2–T0)	−0.70 (2.12)	−0.38 (4.27)	−0.68 (2.81)
GRCS_IS Change score (T2–T0)	−0.78 (2.88)	−0.19 (4.49)	0.58 (3.67)
GRCS_IC change score (T2–T0)	1.17 (3.04)	−0.88 (2.19)	0.63 (2.09)
GRCS_PC change score (T2–T0)	−1.87 (5.20)	−1.06 (5.89)	−0.84 (4.75)
GRCS_IB change score (T2–T0)	−1.48 (3.98)	−0.56 (4.03)	−0.63 (4.49)

#### Comparison of “T0–T1” Evolution (Pre-test vs. Immediate Post-test) Between the Experimental Groups and the Control Group According to the Type of Game and the Status of Gamblers

The results are given in Table 2.

**Table 2:** ANOVA results adjusted for the status of gamblers and the type of gambling comparing the 2 experimental conditions with the control condition for differences between the pre-test (T0) and 1st post-test (T1).

	**Effect (*****F*** **and** ***p*****-value)**						
	**Pop-up messages**		**Type of game**		**Status of gambler**		**Pairwise comparisons for significant effects with Dunnett's tests^**‡**^**
	***F***	***p*-value**	***F***	***p*-value**	***F***	***p*-value**	
Money wagered	4.41	**0.0170**	2.73	0.1043	0.80	0.3766	a^NS^, b^NS^
Time spent	1.36	0.2845	61.69	** <0.0001**	0.60	0.4426	NA
Enjoyment of gambling	0.70	0.5015	0.15	0.7009	3.37	0.0721	NA
GRCS_GE change score	0.55	0.5813	0.91	0.3434	0.01	0.9123	NA
GRCS_IS change score	0.18	0.8352	1.63	0.2078	0.86	0.3570	NA
GRCS_IC change score	1.81	0.1736	0.00	0.9643	0.92	0.3427	NA
GRCS_PC change score	0.61	0.5446	1.01	0.3186	1.70	0.1983	NA
GRCS_IB change score	0.31	0.7379	0.66	0.4188	0.23	0.6336	NA

*NA, not applicable (no effect of the pop-up condition or of the interactions)*.

‡ *a, self-appraisal pop-up vs. control; b, informative pop-up vs. control; NS, non-significant*.

*p-values indicated in bold are those under 0.05 (level of statistical significance)*.

We did not demonstrate any significant differences between the experimental groups (self-appraisal and informative pop-ups) and the control group regarding all outcomes. A significant effect of pop-up messages on money wagered was identified (*p* = 0.0170) but pairwise tests showed that money wagered did not differ between self-appraisal condition vs. control group (*p* = 0.2430) and between informative condition vs. control group (*p* = 0.1966). Time spent gambling was significantly different between skill and chance bank games and skill and chance social games (*p* < 0.0001).

#### Comparison of “T0–T2” Evolution (Pre-test Vs. 2nd Post-test) Between the Experimental Group and the Control Group According to the Type of Game and the Status of Gamblers

The results are shown in Table 3.

**Table 3:** ANOVA results adjusted for the status of gamblers and the type of gambling comparing the 2 experimental conditions with the control condition for differences between the pre-test (T0) and 2nd post-test (T2).

	**Effect (*****F*** **and** ***p*****-value)**						
	**Pop-up messages**		**Type of game**		**Status of gambler**		**Pairwise comparisons for significant effects with Dunnett's tests**^**‡**^
	***F***	***p*****-value**	***F***	***p*****-value**	***F***	***p*****-value**	
Money wagered	1.63	0.2076	2.83	0.0996	0.33	0.5673	NA
Time spent	1.26	0.2926	26.27	** <0.0001**	2.54	0.1179	a^***^, b^NS^
GACS-Anticipation	0.82	0.4441	0.12	0.7305	0.00	0.9837	NA
GACS-Desire	0.84	0.4360	0.99	0.3241	2.02	0.1606	NA
GACS-Relief	0.95	0.3945	0.63	0.4304	0.10	0.7516	NA
**GRCS_GE** ** change score**	0.05	0.9549	0.04	0.8349	3.46	0.0687	a^*^, b^NS^
GRCS_IS change score	0.94	0.3971	1.33	0.2546	3.36	0.0723	NA
**GRCS_IC** ** change score**	4.44	**0.0167**	0.14	0.7076	0.45	0.5035	a^NS^, b^***^
GRCS_PC change score	0.21	0.8101	0.01	0.9224	0.22	0.6438	NA
GRCS_IB change score	0.32	0.7269	0.00	0.9771	1.08	0.3030	NA

*NA, not applicable (no effect of the pop-up condition or of the interactions)*.

‡ *a, self-appraisal pop-up vs. control; b, informative pop-up vs. control; NS, non-significant*.

* *p < 0.05;*

*** *p < 0.001*.

*p-values indicated in bold are those under 0.05 (level of statistical significance)*.

No significant difference between the experimental groups and the control group regarding the evolution of money wagered was found. A significant effect of the interaction of pop-up messages on time spent gambling for skill and chance bank gamblers was demonstrated, with a lower duration in self-appraisal condition compared to control condition (*p* = 0.0078). Rregarding the secondary outcomes, a significant effect of the interaction of self-appraisal condition on the score of the GRCS-GE compared to the control group for skill and chance bank gamblers was also demonstrated (*p* = 0.0463). In the control condition, the change score of the GRCS-GE was negative (mean value: −1.08), which indicated that gambling expectancies decreased in comparison of pre-test score. In the self-appraisal condition, the mean value increased in comparison of pre-test score (mean value: +0.95). The results revealed that participants in the pop-up conditions and the control condition significantly differed in the illusion of control dimension of the GRCS (GRCS-IC) according to gambler status. The comparisons revealed a significant decrease in the GRCS-IC for ARGs in the informative pop-up condition (mean value: −1.86) compared to an increase in the control condition (mean value: +2.70) (*p* = 0.0072).

### Description of Recall and Display Time of Pop-Up Messages

The display time of the pop-up message on the screen before it was closed by the participant varied according to the condition (Table 4). We observed a rapid decrease in the display time of pop-ups in the control group, while this display time was more important and stable in the informative condition and in the self-appraisal condition despite a slight decrease after the second pop-up.

**Table 4:** The mean display time, in seconds, of the pop-up messages according to the experimental condition.

	**Display time** ** POP-UP 1**	**Display time** ** POP-UP 2**	**Display time ** **POP-UP 3**	**Display time** ** POP-UP 4**
Control condition	6.47	6.69	4.69	3.80
Experimental condition informative pop-up	8.67	6.57	8.12	8.46
Experimental condition self-appraisal pop-up	8.55	6.51	7.53	7.77

At the end of the experiment, we asked the participants to recall the content of the 4 pop-up messages displayed during their gambling session. We observed better free recall of the content for self-appraisal messages than for informative messages. Thus, 77% of the participants in the self-appraisal condition recalled one pop-up message correctly, while only 35% of the participants in the informative condition recalled one pop-up message correctly. Beyond the first message recalled correctly, the majority of participants failed to remember a second message correctly (32% in the self-appraisal group vs. 12% in the informative group). Similar results were observed at the post-test 15 days after the experiment.

For all participants (no difference between ARG and LR/NPG), the most relevant informative message was “*Gambling involves risks: debt, loneliness, and addiction*.” According to the participants, the most relevant self-appraisal message was “*Do you spend more money than you expected?*.”

### Qualitative Data Analysis

Finally, the majority of participants (83%) in the experimental groups said that the pop-ups did not have any impact, or only a low impact, on their gambling practice during the session. This proportion was higher in the group of ARGs (94%). In addition, 53.6% of participants declared they were disturbed by the pop-ups during their gambling session. Similarly, we observed that this proportion was higher in ARGs (68.7%). Participants explained that pop-ups were “*irritating*” and “*frustrating*” because they “*associated them with pop-ups ads*.” Pop-ups “*disturbed their gambling practice*” and “*distracted*” them.

Despite these negative aspects, the majority of experimental group participants (64.7% of ARG vs. 80% of LR/NPG) thought that the pop-up messages could be useful for protecting players and reducing the risks of excessive gambling. They explained that pop-up messages “*can make you think”* and “*could make people aware of the risks*.” They allow gamblers “*to be informed*” and to “*remain vigilant*.”

## Discussion

The present study investigated the effect of Internet gambling-related pop-up warning messages (self-appraisal and informative messages) compared to that of a control condition on gambling behavior, experience of play, recall of messages, display time of messages, cognitive distortions, and craving, taking into account the type of game and the severity of the participant's gambling practice. According to the literature, we hypothesized that self-appraisal messages would have a larger effect than informative or control conditions. We also hypothesized that the impact would differ according to the gambler's characteristics (status and type of preferred game). The results contradicted our expectations because we observed no significant differences between the three conditions at the end of the gambling session in the quantitative data (gambling behavior, cognitive distortions, and craving).

However, follow-up demonstrated an effect of self-appraisal pop-ups compared to control group with a decrease on time spent gambling (*p* = 0.0078), but only for sport and horse bettors. This result supports the idea that self-appraisal messages can help gamblers change their gambling behavior and that this effect varies according to gamblers profiles such as favorite gambling-type (25).

Moreover, as demonstrated in the literature, warning messages might help to correct some gambling-related cognitive distortions (22, 36–38). Indeed, we observed a significant decrease of the illusion of control (GRCS-IC) for ARGs in the informative pop-up condition compared to the control condition (*p* = 0.0072). A reminder of the risks associated with gambling appears to modify gambling-related thoughts in the short term. In contrast to the study of Monaghan (27), we observed that informative messages had a larger influence on reducing cognitive distortions than self-appraisal messages for ARGs. As a consequence, informative messages could also be a useful way to prevent the risks associated with gambling and to facilitate responsible gambling. Moreover, we observed that this influence was more important for ARGs than for LR/NPG. Low risk/non-problem participants gambled within appropriate levels and may not be concerned by this kind of message. This reaction may explain the lack of impact observed. The impact on illusion of control demonstrated that early intervention for ARGs with appropriate messages could decrease the effect of the game on gamblers' cognitions and may secondarily limit the transition to excessive practice. However, the effect of the warning messages on the reduction of cognitive distortions must be put into perspective. While pop-up messages seem to have a positive impact on the illusion of control, no significant effect on other types of cognitive distortions was observed for ARGs. We even observed an increase of gambling-related expectancies (GRCS-GE) (*p* = 0.0463) for sport and horse bettors with self-appraisal pop-ups condition compared to control condition.

On the other hand, as suggested by Gainsbury et al. (18), the results showed that appropriate recall is not necessary to modify gamblers' thoughts. In fact, despite participants remembering self-appraisal messages better, we only observed an impact of informative messages on cognitive distortions. Moreover, if this result suggested that participants were paying better attention to the self-appraisal messages' content, we observed that, beyond the first message recalled correctly, the majority of participants failed to remember a second message correctly. In accordance with the participants' comments collected after the experiment and as underlined by the study of Hollingshead et al. (28), the results suggested that many gamblers did not pay attention to the pop-up message content, especially in the case of successive pop-ups. Additionally, in contrast to the experiment of Cloutier et al. (39), which found that wagering decreased when players were forced to pause their gambling session for 7 s, and in contrast to Stewart and Wohl (40), which showed that pop-up messages that interrupt gambling sessions reduced dissociation, our results demonstrated that a pop-up message display time longer than 7 s did not impact gambling behavior. It is possible that gamblers did not pay attention to the warning information because they believed they were in control of their gambling behavior and because they knew exactly how much they were spending and how long they had played (41, 42). Consequently, the information provided in the pop-up message may have been redundant with the information they thought they already had (28). In fact, 90% of the participants in our study reported that both time spent and money wagered were appropriate according to their means, and only 14% of the sample felt they had a problem with their gambling during the experiment.

It is important to note that, in contrast to Gainsbury (18), in which only a minority of participants indicated that the messages had reduced their enjoyment (12.2% of informative and 11.7% of self-appraisal groups), we observed that a majority of the participants (53.6%) in our study were disturbed by the pop-ups during their gambling session. This proportion was higher in ARGs (68.7%). The majority of previous studies took place in a laboratory with an EGM simulator (16), while our study targeted Internet gamblers. Users on the Internet generally receive pop-up messages every time they initiate a search on the Internet or activate a new app or programme, for example. A consequence of the excessive frequency of pop-up messages on the Internet is that their content is ignored (43), and increasing the amount of information in the pop-up does not increase attention to the content (28, 44). Thus, Internet gamblers may process gambling pop-ups as they usually process each pop-up received on the Internet. This assumption is supported by the fact that gamblers from the two experimental groups declared that they associated gambling pop-ups to pop-up ads and that this association increased their irritation and frustration. This feeling was probably increased in ARGs because the involvement and the immersion in the game were more important to them than it was to other gamblers. In general, immersion and dissociation are particularly important for Internet gambling, in contrast to studies conducted with EGM simulators in the laboratory (4, 45). Similarly, as demonstrated by Blaszczynski, breaks in play could increase frustration (23). However, we did not observe an effect on enjoyment, probably because breaks in play were accompanied by warning messages. Last, it is possible that this frustration was caused by the excessive frequency of the pop-ups. It could be interesting to test Internet pop-ups with varying frequencies.

Several limitations that could influence the results must be highlighted. Our sample was small, which could lead to a lack of power to detect significant differences between conditions. Therefore, it might be interesting to replicate this study with more participants. Moreover, even though we tried to create an ecological gambling experience, the sessions were conducted in a hospital under experimental conditions. These divergences from a real gambling environment (laboratory being less comfortable than home, impacting immersion) could have influenced the results. For example, in order to facilitate the data collection participants could only play on their favorite website. Even if a majority of the participants were registered on 1 gambling website (73%) or 2 (18%), this obligation could have influenced the gambling session. However, the laboratory design of our study enabled us to have access to gambling experience through face-to-face interviews, which gave us access to richer data, especially regarding subjective gambling experience. Finally, comparisons were limited because of the absence of problem gamblers (not included for obvious ethical reasons) and the exclusion of pure chance gamblers. Moreover, we choose to combine low-risk and non-problem gamblers while a number of them experimented harm related to their gambling. Although a recent study of (46) demonstrated that “PGSI categories are quite sensitive and higher risk PGSI groups consistently reported more harms and more serious harms than lower risk groups.” Nevertheless, the results might be different if we separated our groups differently. It could be interesting to observe the impact of the moderator on problem gamblers, particularly concerning the impact on cognitive distortions and level of disruption. The exclusion of slot machine, scratch ticket, and lottery gamblers could partly explain why we observed few differences between different types of games. Nevertheless, we obtained high ecological validity (participant's favorite gambling website, with her/his own gambling account and with her/his own money, with instructions that they could stop at any time), which increases the authenticity of the participants' responses to the messages and their impact (47). The methodology of the MOD&JEU study was robust, and our results enhance the current knowledge in the literature with the many quantitative and qualitative data collected.

## Conclusions

The results of our study demonstrated the limited impact of pop-up warning messages on gambling behavior and cognition in Internet gamblers according to the type of game and the status of the gamblers. We can make the assumption that a behavioral change takes place over a long period of time, so we need research to assess the longer-term impacts of pop-up messages on time spent and money wagered. Nevertheless, the limited impact of messages on gambling behavior and the inconvenience of the pop-ups for Internet gamblers lead us to only consider warning messages as one piece of a larger responsible gambling strategy. The use of pop-ups to inform Internet gamblers about gambling risks may not be the most appropriate strategy because of the excessive frequency of pop-up messages on the Internet, which leads gamblers to ignore the content of the messages and to be irritated by them. Moreover, many gambling websites operate across many jurisdictions; it would therefore be difficult to regulate the implementation and operation of this measure. Other modalities for warning messages on the Internet must be evaluated to prevent the risks associated with excessive gambling. It may be beneficial for researchers to explore other ways to inform gamblers about their practice, with the use of more personalized tools suitable for the preferred type of game and the status of the gamblers.

## Data Availability Statement

The datasets presented in this article are not readily available because the raw anonymised data will be made available only if the purpose is consistent with the consent given by participants and in accordance with the legislation in force in France. Requests to access the datasets should be directed to Gaëlle Challet, gaelle.bouju@chu-nantes.fr.

## Ethics Statement

This study involved human participants, and was reviewed and approved by French Research Ethics Committee (CPP) on January 8, 2013. The participants provided their written informed consent to participate in this study.

## Author Contributions

JL and AS helped to collect the data. JC, GC-B, and MP analyzed and interpreted the data. JC wrote the manuscript with the help of MG-B and GC-B. All authors read and approved the final manuscript.

## Conflict of Interest

JC, MG-B, AS, JL, and GC-B declare that the University Hospital of Nantes have received funding from gambling industry [Française des Jeux (FDJ) and Pari Mutuel Urbain (PMU)] in the form of a philanthropic sponsorship (donations that do not assign the purpose of use). Scientific independence toward gambling industry operators is warranted. The remaining author declares that the research was conducted in the absence of any commercial or financial relationships that could be construed as a potential conflict of interest.

## Publisher's Note

All claims expressed in this article are solely those of the authors and do not necessarily represent those of their affiliated organizations, or those of the publisher, the editors and the reviewers. Any product that may be evaluated in this article, or claim that may be made by its manufacturer, is not guaranteed or endorsed by the publisher.
